# Crucial Contributions: A Biocultural Study of Grandmothering During the Perinatal Period

**DOI:** 10.1007/s12110-019-09356-2

**Published:** 2019-12-04

**Authors:** Brooke A. Scelza, Katie Hinde

**Affiliations:** 1 Department of Anthropology, UCLA, Los Angeles, CA 90095-1553 USA; 2 Center for Behavior, Evolution and Culture, UCLA, Los Angeles, CA 90095-1553 USA; 3 School of Human Evolution and Social Change, Arizona State University, Tempe, AZ 85287 USA; 4 Center for Evolution and Medicine, Arizona State University, Tempe, AZ 85287 USA

**Keywords:** Cooperative breeding, Breastfeeding, Grandmothers, Maternal and child health

## Abstract

Maternal grandmothers play a key role in allomaternal care, directly caring for and provisioning their grandchildren as well as helping their daughters with household chores and productive labor. Previous studies have investigated these contributions across a broad time period, from infancy through toddlerhood. Here, we extend and refine the grandmothering literature to investigate the perinatal period as a critical window for grandmaternal contributions. We propose that mother-daughter co-residence during this period affords targeted grandmaternal effort during a period of heightened vulnerability and appreciable impact. We conducted two focus groups and 37 semi-structured interviews with Himba women. Interviews focused on experiences from their first and, if applicable, their most recent birth and included information on social support, domains of teaching and learning, and infant feeding practices. Our qualitative findings reveal three domains in which grandmothers contribute: learning to mother, breastfeeding support, and postnatal health and well-being. We show that informational, emotional, and instrumental support provided to new mothers and their neonates during the perinatal period can aid in the establishment of the mother-infant bond, buffer maternal energy balance, and improve nutritional outcomes for infants. These findings demonstrate that the role of grandmother can be crucial, even when alloparenting is common and breastfeeding is frequent and highly visible. Situated within the broader anthropological and clinical literature, these findings substantiate the claim that humans have evolved in an adaptive sociocultural perinatal complex in which grandmothers provide significant contributions to the health and well-being of their reproductive-age daughters and grandchildren.

It is midafternoon when we arrive at the compound. All of the women and children in camp are sitting together in the shade outside of one of the huts. As we begin to update our census to determine household composition, we learn that there is a woman inside the hut who had given birth the night before. I peek inside to say hello and then leave her to rest. The woman’s mother is sitting outside the hut cooking a maize meal porridge. Her daughter has been here for about a month, she tells us, and will stay for several more, while she recovers from the birth.

A few weeks later an older woman arrives at our camp, carrying her grandchild, a tiny infant, in her arms. She unwraps the baby and explains that since her birth the night before the infant has been struggling to breathe. She has come to us because she knows we have a vehicle and can get the baby to a clinic 10 miles away.^1^

The work of these women, in the first case subtle but enduring and in the second vital and acute, represents the critical role that grandmothers play during the perinatal period. We know from decades of research that grandmothers are key providers of allomaternal care (Hrdy 2005; Kramer 2010). A suite of behavioral studies has detailed the ways in which grandmothers aid in provisioning and caring for their grandchildren (Crittenden and Marlowe 2008; Gibson and Mace 2005; Kaptijn et al. 2010; Meehan et al. 2013; Perry 2017; Scelza 2009), while broader cross-cultural reviews have demonstrated the consistent and positive impact that grandmothers, particularly maternal grandmothers, have on child survival (Sear and Mace 2008; Fox et al. 2010).

Despite the richness of this literature, our knowledge about the proximate mechanisms that lead to correlations between grandmaternal support and maternal and infant health remains incomplete. Observational studies provide clues, citing assistance with household chores and productive labor (Gibson and Mace 2005; Leonetti et al. 2005; Meehan et al. 2013) and the importance of supplemental provisioning (Hawkes et al. 1997; Hooper et al. 2015). Most studies, however, investigate grandmaternal contributions to mothers and babies across an extended period, from infancy through toddlerhood or beyond. Here, we focus on grandmothering during one particular time period, the perinatal, which remains largely understudied in the cooperative breeding literature, and which we posit provides important clues to understanding why grandmothers’ presence is so commonly correlated with improved maternal and infant health outcomes. In particular, we propose that mother-daughter co-residence during the perinatal period serves an important adaptive function. The constant availability of a skilled and devoted caretaker during this critical time is likely to impact the health of both the new mother and her infant. Maternal grandmothers, who are both highly skilled in child-rearing and highly motivated to improve the well-being of their close kin, are ideal allomothers during this period (Hrdy 2005). The greater lifetime reproductive experience of grandmothers, especially postreproductive grandmothers, may afford them greater capacity and knowledge to invest more tactically during critical stages of offspring development (Cameron et al. 2000; Chan et al. 2016).

We posit that grandmothers have a unique and adaptive role within the human perinatal complex. Our assertion is predicated on three assumptions: (1) that instrumental, informational, and emotional perinatal support are important enough to have demonstrative effects on fitness; (2) that grandmothers are willing and able to provide the kinds of care required to support new mothers; and (3) that there is both historical and cross-cultural evidence of mothers and daughters co-residing during the perinatal period, particularly for first births (primiparity). In other words, mothers were available to support their daughters during the perinatal period. We substantiate these assumptions through a review of the literature, followed by a case study of grandmaternal support during the perinatal period derived from a series of semi-structured ethnographic interviews and focus groups with Himba pastoralists residing in northwest Namibia.

## An Adaptive View of Mother-Daughter Support During the Perinatal Period

### Support Is Critical during the Perinatal Period

The days and weeks surrounding a birth are a particularly precarious time for both mother and infant. Maternal mortality rates are highest during labor and childbirth and in the immediate postpartum period (Li et al. 1996; Ronsmans et al. 2006), and the global estimate for the lifetime risk of maternal mortality is 1 in 74 women (Ronsmans et al. 2006). Many of these deaths are preventable, especially with improved hygiene, early intervention, and quality intrapartum and postpartum care. Globally, the neonatal period—the first 28 postnatal days—is the period of greatest vulnerability during childhood and accounts for nearly half of all mortality under age 5 (UN Inter-Agency Group for Child Mortality Estimates [IGME] 2017). In 2016, neonatal mortality accounted for ~ 2.6 million infant deaths worldwide (UN IGME 2017). Although many of these deaths result from congenital defects and complications of preterm birth, a significant fraction are preventable or treatable, such as neonatal tetanus, sepsis, pneumonia, and diarrhea, or occur from intrapartum-related events (UN IGME 2017). Moreover, human infants are born particularly altricial among the Hominidae, requiring extensive direct and indirect care from the mother and a network of allomaternal caregivers (Antón and Josh Snodgrass 2012; Trevathan and Rosenberg 2016).

Help during the perinatal period has value for any birth (Rosenberg and Trevathan 2018), but women giving birth for the first time are likely to particularly benefit from the support of their mother (the maternal grandmother). Among mammals, primiparous females are often characterized by poorer reproductive outcomes due to constrained body condition, behavioral experience, and neurophysiological function (Bridges 2016; Nuñez et al. 2015; Pittet et al. 2017; Snyder et al. 2016). Among humans, in the immediate neonatal period, primparity can be associated with suboptimal infant breastfeeding behavior and delayed onset of lactation (Dewey et al. 2003), heightened maternal anxiety (Hou and Shen 2008), and greater experiences of fatigue (Taylor and Johnson 2013). First-time mothers in particular have a steep learning curve as they transition into the behavioral biology of motherhood, even in cultures where prereproductive allocare is common (Hinde 2015). The early postnatal period is important for mother-infant bonding (Kinsey and Hupcey 2013; Wittkowski et al. 2007) and the establishment of breastfeeding to reduce infant mortality (Dewey et al. 2003; Edmond et al. 2006; Mullany et al. 2008). At the same time that breastfeeding is established, mothers learn how to hold, swaddle, and safely co-sleep with their infant (McKenna and Gettler 2016; McKenna et al. 2007). SIDS and accidental suffocation are both significantly reduced in the context of “breastsleeping” with the use of simple, safe co-sleeping practices such as keeping loose bedding away from babies (Gettler and McKenna 2010; McKenna and Gettler 2016). Insufficient knowledge and emotional support can also lead to serious harm for babies. Poor lactation initiation, limited breastfeeding knowledge, and insufficient milk intake in the neonatal period can cause dehydration (Yaseen et al. 2004), brain damage from abnormally elevated unconjugated bilirubin (severe hyperbilirubinemia), and excess weight loss (Dewey et al. 2003; Gartner 2007). However, almost all of these studies come from so-called WEIRD populations (Henrich et al. 2010). Much less is known about when and how women learn these skills, or more broadly how commonly breastfeeding difficulties or insufficient infant care leads to poor health outcomes in other societies.

New mothers are simultaneously grappling with their own recoveries and the fragile health of their newborns. In humans, the key to coping with this challenge is social support. Extensive research has substantiated the benefits of social support, and the consequences when absent, during the perinatal period. Support during pregnancy, whether by kin or through social services, improves birth outcomes (Feldman et al. 2000; Hoffman and Hatch 1996; Oakley 1985; Oakley et al. 1990; Scelza 2011a), contributes to easier labors (Collins et al. 1993; Sauls 2002; Scott et al. 1999), and reduces infant morbidity and mortality during the first year (Kana’iaupuni et al. 2005; Leonetti et al. 2005; Sherraden and Barrera 1996). These effects are particularly strong among first-time and young mothers (Heins et al. 1987; Lahdenperä et al. 2004; Leonetti et al. 2005). Emotional and instrumental support by spouses and kin is linked to higher rates of breastfeeding initiation and duration (Asiodu et al. 2017; Meedya et al. 2010; Raj and Plichta 1998) and is critical to women as they learn to breastfeed (Warren 2005). Social support also appears to play a key role in reducing the incidence of postpartum depression. In non-Western cultures, where kin support is prominent, postpartum depression is much rarer than it is in the USA and Europe (Bashiri and Spielvogel 1999; Stern and Kruckman 1983). Greater social support also impacts attachment between mother and infant (Crockenberg 1981). More broadly, increased parenting satisfaction is linked to self-efficacy, and self-efficacy is greatly enhanced by consistent support during the postpartum period (Warren 2005).

Social support can additionally influence maternal and infant health by helping women to balance productive and reproductive demands. Reproduction is energetically expensive, with the costs of lactation outweighing those of pregnancy (Dufour and Sauther 2002; Power and Schulkin 2016). The extended period of human development reduces daily energetic costs of pregnancy and lactation, and somatic reserves buffer the maternal-infant dyad from short-term fluctuations in nutritional intake. Therefore, both the length of pregnancy and the length of lactational amenorrhea are sensitive to maternal energy balance (Ellison 2003; Valeggia and Ellison 2009). That balance is a function of a woman’s current condition and the level of energetic stress she experiences (Jasienska 2003; Valeggia and Ellison 2001). For example, changes in ovarian function occur seasonally in concert with variations in women’s workload (Jasienska and Ellison 2004; Panter-Brick and Ellison 1994) and where seasonal food shortages are common (Bailey et al. 1992). Similarly, the metabolic load hypothesis (Ellison 1994; Ellison et al. 1993) proposes that the length of lactational amenorrhea depends on the amount of energetic stress to sustain milk synthesis and breastfeeding in the broader ecological context. Poor nourishment or intense workload during lactation exerts a greater metabolic load, extending postpartum reproductive suppression relative to that of a woman producing similar amounts of milk but who is characterized by better condition with fewer labor demands. Careful empirical work has provided substantial support for this theory in humans (Lunn et al. 1984; Valeggia and Ellison 2004) and more broadly among primates (Emery Thompson 2017). A sustained period of negative energy balance not only can delay time to the next pregnancy (impacting lifetime fertility) but can precipitate maternal depletion syndrome, which is associated with future preterm births, fetal growth retardation, and increased risk of maternal morbidity and mortality (King 2003).

Social support, either through substitute labor or through provisioning, can be critical to improving women’s energy balance. In many cultures, postpartum rituals and taboos function to limit the work that new mothers are allowed to do, and these rituals almost always occur along with help from others, particularly kin, to make up for productive deficits (Eberhard-Gran et al. 2010). A period of 40 days is customary in many cultures, and this time period matches well with the needs of new mothers in terms of postpartum healing (Visness et al. 1997) as well as providing a specific time set aside for rest, learning, establishing breastfeeding, and emotional bonding with the infant and with key allocare providers (Dennis et al. 2007; Raphael 1966).

As infants grow, social support continues to affect maternal lactational output, most commonly by impacting decisions about weaning. One cross-cultural study showed that the availability of alloparental care was associated with earlier age at weaning (Quinlan and Quinlan 2008). The authors surmise that this association is due to the fact that kin often provide childcare, allowing mothers to resume productive activities away from the home (and their infants) earlier. Similarly, in contexts where female wage labor is prominent and where infant formula is widely available, instrumental support can reduce breastfeeding and precipitate younger weaning age because kin support facilitates mothers’ return to work earlier (Emmott and Mace 2015). Support from spouses, however, facilitates sustained lactation and later weaning (Quinlan and Quinlan 2008). Similarly, among Hadza, women’s production is lower and is subsidized by increases in their husband’s production, allowing mothers to prioritize infant care and feeding (Marlowe 2003). A woman’s elder children can contribute to household subsistence that allows a mother to increase overall fertility (Kramer 2010). Worldwide, evidence of postpartum rituals that prevent women from participating in productive labor or household chores are common (Raphael 1966), so the ways in which social support impacts women’s ability to balance production and reproduction are very much a product of local context and the kinds of support they have available. But what is universal is that women rely on others to directly and indirectly help raise their children.

### Maternal Grandmothers Fill a Unique Niche in the Perinatal Support Structure

Among the suite of potential helpers in the human cooperative breeding system, grandmothers, particularly maternal grandmothers, have been identified as playing an influential role, prompting Sarah Hrdy (2005) to refer to them as the “ace in the hole” among a suite of helpers. In a study of perinatal support in urban India, the role of a woman’s mother is described as follows:

If there was one universal theme that emerged out of all the interviews, it was the reliance that women placed on their own mothers (Amma). Women relied on their mothers for support, advice, for their presence, their help. . . . The mother was seen as a constant, someone women relied on for everyday succor or advice throughout the perinatal period, but also someone who was the essential support through the whole delivery process (Raman et al. 2014).

This sentiment is representative of a global trend in allocare emphasizing the role of the maternal grandmother. In her 1966 dissertation, Dana Raphael evaluated the Human Relations Area Files and determined that the “mother’s mother” is the most typically identified individual assisting during the perinatal period. Across traditional cultures for which information was available, women most typically return to their mother’s home to deliver (42 of 83 cultures), have their mother present at the delivery (54 of 114 cultures), and mother’s mother is the most likely individual to support the mother during the days and weeks of postnatal seclusion (Raphael 1966). Here we describe how maternal grandmothers provide three critical forms of support: instrumental, informational, and emotional.

Instrumental support by grandmothers can reduce trade-offs for mothers between productive and reproductive efforts. This can occur when grandmothers provide either direct infant care, allowing mothers more time to devote to production, or indirectly through labor substitution, which allows mothers to spend more uninterrupted time with their infants while still having their caloric demands met. Alternatively, instrumental support can reduce trade-offs between reproduction and maintenance (Kramer and Ellison 2010). Grandmothers who help with infant care, cooking, or other household tasks can free up mothers to rest and recuperate after a birth, or to slow down her activities in the period preceding birth. Here we discuss in more detail the benefits that can come from grandmaternal provisioning of food, direct care, and labor during the perinatal period.

Discussion of provisioning by postmenopausal women in the evolutionary anthropology literature has tended to focus on providing supplemental food to infants and toddlers (Fouts and Brookshire 2009; Hawkes et al. 1997; Meehan and Roulette 2013). In the perinatal period, provisioning would more typically involve feeding the mother. Providing food for new mothers is commonly mentioned in ethnographic studies of cross-cultural birth practices, and a woman’s mother is often in charge of this care (Eberhard-Gran et al. 2010). Postpartum provisioning is said to help the mother regain her strength and improve breastfeeding, and it involves both consumption of particular, often protein-rich foods and eating more meals per day (Pillsbury 1978; Raven et al. 2007). In China, the period of *zuo yuezi* or “doing the month” allows mothers 30–40 days to recover after a birth, with considerable support from her mother or mother-in-law (Holroyd et al. 1997; Pillsbury 1978). In Nigeria, new mothers are placed in a “fattening room” for two to three months after birth. There they are cared for most often by their mothers (or, when she is unavailable, by their mothers-in-law), and her only job is to rest, eat, and feed her baby (Kelly 1967). Women’s mothers are also often in charge of preparing particular food and drinks that are thought to help with postpartum healing.

Grandmothers have also exhibited a special role in providing direct infant care, including feeding, bathing, and soothing (Crittenden and Marlowe 2008; Leonetti et al. 2005; Meehan 2005; Scelza 2009). Among the Martu, an Aboriginal group living in Australia’s Western Desert, grandmothers not only provide significant amounts of direct care, they provided that care strategically. Martu grandmothers tended to do more high-skill, high-demand care such as feeding, bathing, and soothing infants when they were distressed, and relatively less low-cost, low-skill care such as holding babies, which could be done by any number of helpers (Scelza 2009). Such care is particularly important after a first birth, when women’s learning curve is the steepest, and in the first few weeks after a birth, when mistakes can be particularly costly (Warren 2005). In many other contexts, a woman’s mother is a major source of support with infant care and caring for older children after a birth (Crittenden and Marlowe 2008; Dennis et al. 2007), which facilitates the care of multiple dependent offspring that characterizes human reproductive scheduling (Gurven and Walker 2006).

Labor substitution is another arena where women often rely on their mothers after a birth. In some societies, this is quite formalized, as with *zuo yuezi* in China. During this time, women are supposed to stay inside and in bed as much as possible, and they are barred from performing tasks such as washing clothes or dishes. Traditionally, because of patrilocal residence, it is the mother-in-law who is supposed to provide assistance during this time, although more recently, as neolocality becomes more common, women’s mothers are increasingly taking over the burden (Pillsbury 1978). Sequestering mothers after a birth with care from female kin is also seen in sub-Saharan Africa (Ngunyulu and Mulaudzi 2009), South America (Piperata 2008), and South Asia (Choudhry 1997), and a broad cross-cultural overview of the practice suggests that it is widespread around the globe (Raphael 1966). Even where mothers are not required to remain in the home after a birth, they may frequently rely on kin to help with household and productive labor when they have young babies (Gibson and Mace 2005; Meehan et al. 2013; Scelza 2011a).

Grandmothers provide informational support, especially during difficulties and challenges. In the perinatal period, informational support most often relates to advice about breastfeeding and complementary feeding, information about infant care and safety, and assistance with diagnosis and treatment of health problems. Outside of the formalized health care system, women’s mothers (and mothers-in-law) are routinely mentioned as a key source of information on all of these topics. Despite the ubiquity of this finding, very little detailed information is available about the specific kinds of information that mothers provide to their daughters or the types of instructional techniques they use in the case of direct teaching. Similarly, there is a rich literature detailing the use of traditional medicine for pregnancy, postpartum recovery, and lactation induction, and older women are largely the holders of this knowledge (see Srithi et al. 2012 for a recent review), but studies focusing on knowledge transfer and the learning process are rare. Although the details remain vague, we do know that mothers play a key role in transferring information. Among the Bataknese in Indonesia, women reported that the making and serving of a medicinal soup believed to serve as a lactogogue (breast milk stimulant) was explicitly passed from mother to daughter (Damanik 2009). In Thailand, mothers and mothers-in-law were responsible for passing on information about postpartum healing practices (Kaewsarn et al. 2003). Although informational support is likely to have the greatest impact for primiparous mothers, since each birth and each infant is different, grandmaternal contributions would have sustained value even into multiparity.

Finally, women receive critical emotional support from their mothers during the perinatal period. In a study of grandparental support for babies in the NICU, emotional support was listed by doctors and nurses, and by new parents, as being the most critical type of support they could provide (McHaffie 1992). In the early postpartum period, support from a woman’s mother relieves stress and anxiety (Engle et al. 1990) and is related to greater satisfaction with parenting and competency during the child’s infancy (Hess et al. 2002; Oberlander et al. 2007). Women who currently have positive relationships with their own mothers are characterized as more sensitive to their own infants (Kretchmar and Jacobvitz 2002). Emotional support can also be instrumental for maternal and infant health (Rosenberg and Trevathan 2018). Women who reported having greater emotional support during pregnancy (which came mainly from their own mothers) delivered heavier infants (Campos et al. 2008; Scelza 2011a). In a conventional Western obstetric unit, a randomized control trial demonstrated that the presence of emotional support during labor shortened parturition time and reduced Cesarean section deliveries (Kennell et al. 1991). Social support was associated with reduced maternal fatigue 6 months after parturition (Taylor and Johnson 2013) and improved breastfeeding practices (Asiodu et al. 2017). Among Puerto Rican women living on the mainland and in Puerto Rico, women who were both emotionally bonded and geographically close to their mothers had better birth outcomes. Even more interestingly, women who were geographically close to their mothers but were not relying on them for social support had worse birth outcomes than women in any other group (Scelza 2011a). Taken collectively, such findings substantiate that humans have evolved within a sociocultural adaptive perinatal complex in which grandmothers likely provide particularly crucial contributions to the health and well-being of their reproductive-aged daughters and grandbabies.

### Grandmothers Were Available to Provide Postpartum Support

Thus far, we have emphasized the role of the maternal grandmother, and the mother-daughter relationship, when describing the perinatal support structure. However, to provide this support, women and their mothers must be in close proximity around the time of a birth. Whereas older cross-cultural analyses of human postmarital residence among foragers tended to emphasize the predominance of patrilocality (Ember 1978), more recent studies have shown that multilocal residence was the modal pattern (Alvarez 2004; Hill et al. 2011; Marlowe 2004; Scelza and Bliege Bird 2008). When multilocality occurs, couples typically live with the bride’s family in the early years of marriage, which, along with allowing for the practice of brideservice, provides women the opportunity to continue to rely on her parents for support in the matrimonial and motherhood transitions (Harrell 2018). This sets up the possibility for extended perinatal support for early births. Among horticulturalists, similar patterns emerge; women tend to reside with kin, particularly their parents, more often than do men (Walker et al. 2012).

Pastoralists and agriculturalists exhibit quite different patterns. Strict patrilocality is the normative pattern (Borgerhoff Mulder et al. 2010; Shenk et al. 2010). This means that women must either rely on affinal kin for support or find alternative ways to interact with their own kin after marriage. Although help from the mother-in-law is not uncommon (Forman et al. 1990; Leonetti et al. 2005; Symonds 1996), there is also substantial evidence that women in patrilocal societies continue to receive support from their mothers after marriage. Ethnographic accounts of birth often describe visitation patterns that bring mothers and daughters back together. In Japan, this custom, called *satogaeri bunben* or “home to the village to give birth,” requires women to return home during their third trimester of pregnancy and stay through the early postpartum period (Yoshida et al. 1997). In Fiji, where the Indo-Fijian population is patrilocal, women return to their natal homes to be with their mothers during the third trimester of pregnancy and remain there until six to eight weeks postpartum (Morse 1984). In Zimbabwe, primiparous women return home during the third trimester and stay through the birth and puerperium period (Mutambirwa 1985).

Shorter-term help has also been documented, through targeted visits. In some cases, women are visited by their mothers. Among Bedouin Arabs, during the mandated 40-day seclusion period following a birth, women are visited frequently by their female kin, who bring food and gifts and help to relieve the new mother of her chores and help to care for the infant (Forman et al. 1990). Similarly, among the Tsimane, older women with fewer dependent offspring were more likely to travel to visit relatives, indicating a pathway for mother-to-adult-daughter visits (Miner et al. 2014). Gibson and Mace (2005) show that mothers are more likely to visit their daughters than their sons, even if visiting their daughters required travelling to another village. In other cases, though, it was the daughters who reached out to their mothers. For example, in Bangladesh, women with dependent children frequently visit their own mothers (Perry 2017).

To highlight the differences between living with mothers and with mothers-in-law, Leonetti and colleagues conducted a cross-cultural study of the Bengali, where women reside with their husband’s family, and the Khasi, where women remain close to their own kin, including their mothers (Leonetti et al. 2005). Bengali women had a faster pace of reproduction when the mother-in-law was present, and care was provided to the child to buffer against the risks of a faster birth schedule. Among the Khasi, help was provided more directly to the new mother, who received assistance with domestic labor. Khasi women’s birth intervals were longer, but grandmother presence was significantly associated with reduced infant mortality, especially for lower parities, and overall both infant and child mortality were lower among the Khasi than the Bengali. This suggests that whereas paternal grandmothers are invested primarily in the health and well-being of their grandchildren, maternal grandmothers are motivated to help both their daughters and their grandchildren. Other studies have similarly shown that power dynamics between women and their mothers-in-law can result in negative perinatal outcomes (Simkhada et al. 2010; White et al. 2013). In a study of Chinese women who practiced *zuo yuezi*, those who had support from their mother-in-law had double the odds of experiencing postpartum depression relative to women who were supported by their own mothers (Wan et al. 2009). These findings, in concordance with the general pattern that residential flexibility often leads to mother-daughter co-residence around the time of birth, further support the notion that one’s own mother is a particularly vital source of support during the perinatal period.

## Perinatal Support among Himba Pastoralists

The Himba reside in the northwest corner of Namibia, in an area called the Kaokoveld. The environment is semi-arid and mountainous, to which Himba have adapted with semi-nomadic pastoralism that spreads livestock across locations and further decentralizes wealth through cattle lending (Bollig 2006). In some areas, including the study area of Omuhonga, women grow gardens, primarily of maize, sorghum, and melons, to supplement their traditional diet of milk and meat. Women’s work thus consists of tasks related to dairying (e.g., milking, souring and culturing, and occasionally herding) and horticulture (e.g., planting, harvesting, and processing), in addition to everyday tasks such as collecting water and firewood. Much of this work is communal within the household, and women rely on both their natal kin and their co-wives for support (Scelza 2015).

Integration with the market economy remains limited in Omuhonga. Himba patterns of residence, mobility, and economic productivity have been affected by colonial policies from Germany, Portugal (via Angola), and South Africa, as well as current government restrictions that went into place after independence in 1990. For much of the twentieth century, economic diversification was greater than exists today; more recent veterinary regulations limit the ability of local herders to participate in the trade of livestock, and limited education precludes extensive participation in other economic sectors, leading to increased marginalization and reliance on subsistence herding (Bollig 1998, 2013).

Postmarital residence is patrilocal, but the distance between women’s natal and marital homes is typically within a day’s walk. Women regularly visit with their parents and siblings. Among the Himba, it is a widely practiced norm for women to return to their natal compounds late in their pregnancies and remain there through the birth and for some time afterward (Scelza 2011b). Younger children (< 5 years) often accompany their mothers on these visits, while older children who provide critical labor tend to remain behind where they are cared for by their fathers and other kin. Himba women grow up participating in allocare for younger siblings and cousins. Breastfeeding takes place openly and on demand, but early supplementation (within the first 6 months) with goat’s milk and a maize-meal porridge is common. Colostrum taboos, observed widely across cultures (Hinde 2017), are practiced among the Himba. Colostrum is typically withheld, with boiled water or goatmilk substituted during the first two neonatal days. Cosleeping, or rather breastsleeping (McKenna and Gettler 2016), is the norm. Himba sleep on a cow hide substrate on a packed earthen floor with blankets used during the winter months.

Births continue to take place mainly at home, with the assistance of female kin and, when needed, other women who are known to be skilled birth attendants. Previous research on Himba mobility patterns showed that across parities, birthing women had their mothers present 67% of the time (*n* = 428 births), and maternal propinquity for multiparous births remains high (> 60%) through the sixth birth (Scelza 2011b). However, there is a growing trend toward hospital births, particularly for first births, due to a recent public health campaign seeking to increase the rate of medically assisted births. Travelling to the nearest hospital (~ 100 km away) requires substantial financial resources (for travel, food, and lodging) and can mean that women will be separated from their close kin during the perinatal period. These trips can last from a few days to a few months, depending on the availability of transport, the amount of financial support available and whether they are able to have their mothers or other kin accompany them. Since this change was just emerging when our data were collected, and many of the women in our sample were reflecting back to their first births, which took place several years previous, we cannot assess the impacts of this trend with our data.

## Methodology

### Ethnographic Interviews among Himba Women

Using a collective case-study approach, we conducted a series of semi-structured interviews and focus groups to explore the role of grandmaternal support around the time of birth among Himba pastoralists. Collective case-studies, particularly those that combine quantitative and qualitative data, allow for a more holistic investigation of a phenomenon in its natural setting (Crowe et al. 2011). The Himba emphatically maintain their traditional lifestyles and are characterized by many features of reproduction, social organization, and childcare which are thought to have been common throughout human evolutionary history. In addition, because the Himba practice patrilocal postmarital residence, they face some of the residential constraints that were common across human history.

Data for this study were collected in two rounds, in 2013 and 2016. We began our study by conducting a series of unstructured interviews and focus groups with key interlocutors. The first was with a Himba woman who was widely considered one of the best and most competent birth attendants in the area. She was asked questions about pregnancy, birth, breastfeeding, and postpartum care. Four other interviews were held with grandmothers, who shared their experiences helping their daughters during the perinatal period and helped us to understand local social norms about birth and postpartum care. Finally, we held two focus groups, preceding each year’s data collection. The focus groups included women of various ages and parities and prioritized gathering general information about pregnancy, birth, and childcare. These initial interviews and focus groups were used to develop a culturally appropriate interview guide for the semi-structured interviews (Morgan 1996).

### Interview Sample

Semi-structured interviews were conducted with 37 mothers of children less than 3 years of age. Mothers were chosen opportunistically when visiting compounds as part of a larger study on female reproductive decision-making. In 2013, these interviews were combined with the collection of breastmilk as part of a cross-cultural study of infant feeding ecologies (Klein et al. 2017). In addition, some women, having heard about the study, visited our camp in the mornings and evenings and were interviewed there. Women in the sample ranged in age from 15 to 44 (mean = 28.3, SD = 7.8). Average age at first birth was 19.0 years and average parity was 3.8 (Table 1). The majority (81%) of women had more than one child, and 51% of women had more than four children. Women were aged by mapping traditional year names (which refer to events that occurred between rainy seasons) onto Gregorian calendar years (see Scelza 2011b for more details). Infant ages were determined either by viewing health cards or through conversation with the mother to estimate month and year of birth.

**Table 1:** Sample demographics

	Number (%)
Maternal age	
15–19	4 (11.1)
20–29	17 (47.2)
30–39	11 (30.6)
40–49	4 (11.1)
Parity	
1	7 (18.9)
2–4	16 (43.2)
4	14 (37.8)
Age of focal child	
0–6 months	12 (32.4)
7–12 months	12 (32.4)
13–24 months	11 (29.8)
25–36 months	2 (5.4)
Mother’s education	
None	24 (64.9)
1–2 years	9 (24.3)
> 2 years	4 (10.8)

The interviews had several parts. All women were asked about their first birth experience, and, if multiparous, a more limited set of questions was asked about their most recent birth. Covered topics included postpartum health, teaching and learning about infant care and breastfeeding, social support around the time of birth, breastfeeding and complementary feeding, allonursing, and weaning. Finally, reproductive histories, years of schooling, and current marital status were recorded for all women. Here we will focus on the parts of the interview that relate to the perinatal period. Approval for this study was granted by the UCLA Institutional Review Board (#13-000881). Informed consent was obtained from all individual participants included in this study.

## Results

### Omwari: The Period of Perinatal Support

**Fig. 1:**
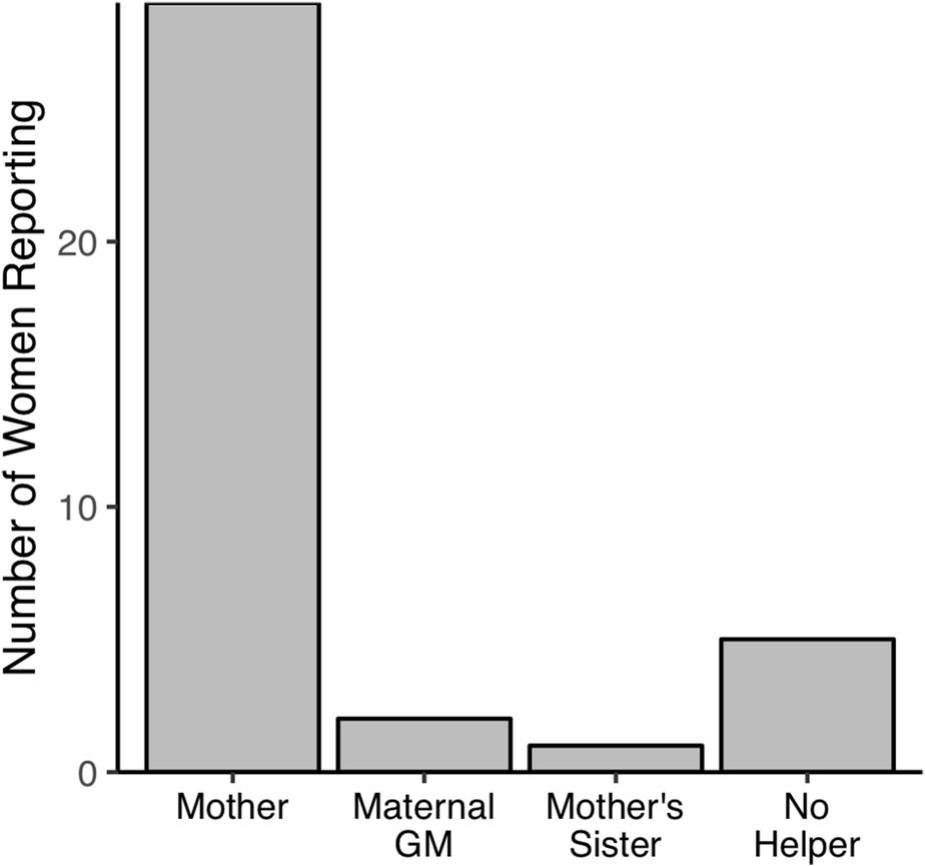
Source of support during the perinatal period surrounding women’s first birth (*n* = 36)

In the Otjihimba language, the period immediately after a birth is called *omwari*. In the present sample, 78% (28 of 36) of women had their mothers present for their first birth, and another three were assisted by other maternal kin (Fig. 1). These numbers are similar to those documented in a much larger sample (400+ births) reported elsewhere (Scelza 2011b). According to the interviews, the period of omwari lasts for 1–2 weeks, ending when the infant’s umbilical cord stump separates. During this time, the new mother is expected to remain in the hut while her mother or other female kin cook and care for her. In addition to the primary goal of rest and recuperation during omwari, this is a period of intensive learning for first-time mothers. A mother (or her surrogate) typically sleeps in the hut with her daughter and the newborn, providing round-the-clock care and advice. Although maternal co-residence typically extends beyond this period, once omwari ends women are expected to resume some of their productive duties.

The length of time that women received postpartum support varied, depending on their location (natal or marital compound), the support network present, their birth experience and recovery, and their household composition (whether a woman had a co-wife who could help with household activities during a perinatal absence). Of these, whether the woman gave birth in her husband’s compound or with her own kin was of paramount importance. One woman explains, “After *omwari* you start cooking yourself and begin your work again. If there is no one there to care for you, you must work again after four days.” A grandmother further explains, “The best is to stay with your mother until the baby is grown [~ 6 months]. If you go back to your compound, you must work. It is good for the mother too because she can keep up relations with her daughter and her daughter can help her with some tasks while she is there, like grinding maize.” The social pressure for women to return to their natal compound as they approach parturition is so strong that there are cultural penalties levied against the husband if a woman gives birth at his home and something goes wrong. If the baby dies, the husband’s family has to pay a fine (typically 1 cow) to the infant’s maternal grandmother.

### Forms of Support

#### Breastfeeding Support and Infant Care

Thirty women were asked about whether they experienced problems learning to breastfeed, and of those 63% (19 of 30) reported some type of difficulty. The most frequently cited problems were difficulty learning to latch and/or pain associated with latching and issues with supply (Table 2).

**Table 2:** Women’s reported problems learning to breastfeed their first child. Multiple responses allowed. Women were only asked in the 2016 portion of the interview sample

Reported difficulty	Number reporting (*n* = 25)
Latch/positioning	19
Supply	15
Pain	8
Fear/anxiety	4
Breast infection (swelling, pain, fever)	4
Infant illness	1
Lack of sleep	1

**Fig. 2:**
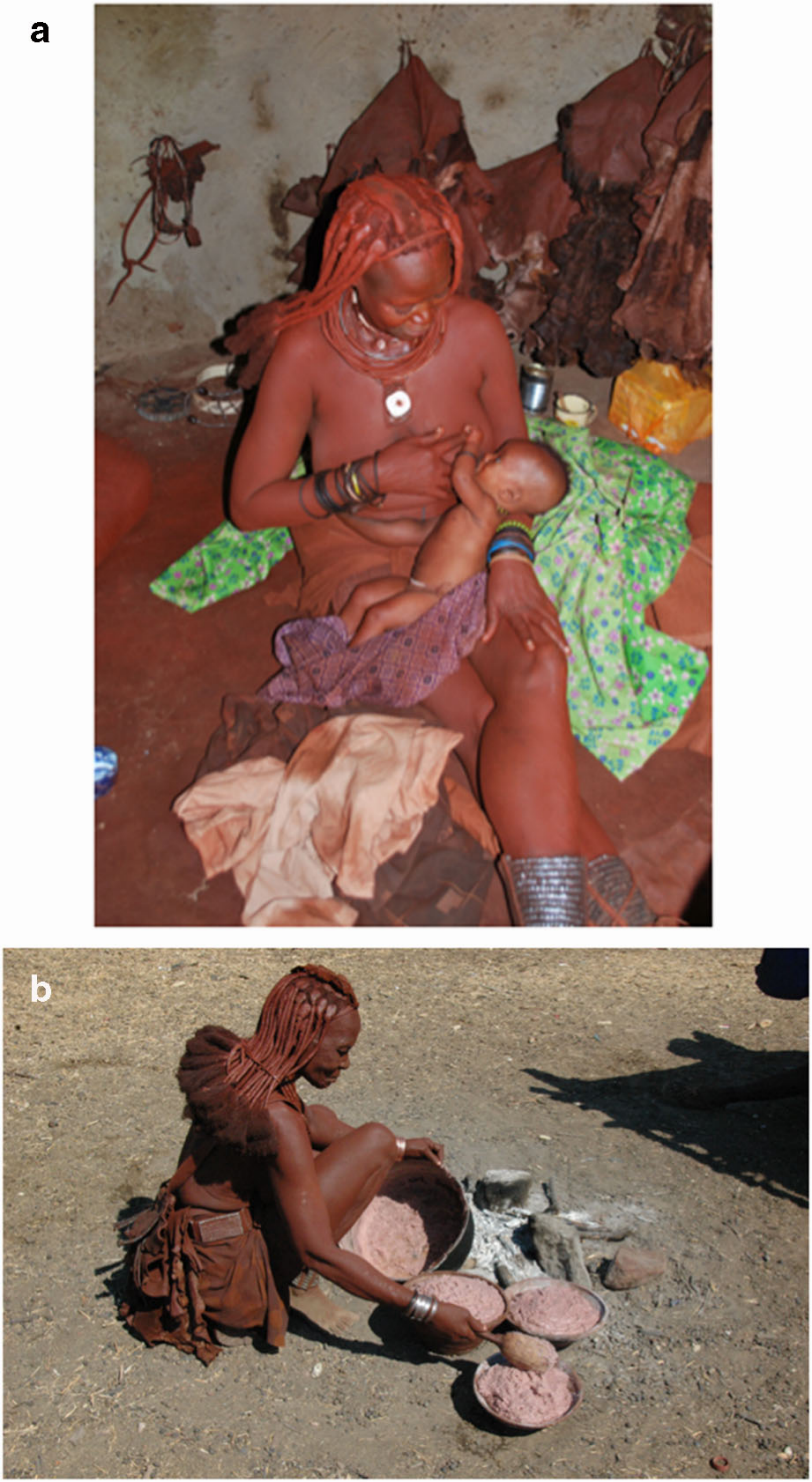
Himba mother at her mother’s home one week after giving birth to her sixth child (top). Grandmother preparing food for her daughter and grandchildren (buttom)

Women received detailed support and advice related to breastfeeding upon giving birth for the first time. In many instances, this included direct teaching (e.g., assisting with infant placement to improve latch). “I didn’t know how to breastfeed, or how to hold him.” “My mom showed me how to put the baby to my breast. She told me to sit, not lie down [to feed]. She told me to feed him four times during the night.” When women struggled, their mothers were there to support them. “When I was scared she [her mother] told me how to get the baby to un-latch without pain.” At times, when gentle support was not enough, women reported that their mothers were more forceful. “I was afraid to do it. I wanted to vomit from the fear. I told my mother that I didn’t want to [breastfeed] and she told me I must do it. At first she told me nicely, but when I still refused then she was yelling at me to do it.” Mothers also provided support consistently. They slept with their daughters in their hut for the first week or more, which meant women had help with every feed, as well as reminders about the importance of breastfeeding regularly. “During the night when the baby would cry she would wake me up and tell me to start breastfeeding,” one woman reported. In a few instances, women reported that they did not have or need help with their first births. “I watched other people before I had kids. I didn’t sleep with my mother. She was far away. I told her don’t bother yourself. Don’t come.” However, this same woman was interviewed for this study at her mother’s compound, where she was residing after her most recent (sixth) birth (Fig. 2).

In addition to learning how to breastfeed, women also relied on their mothers to help them with breastmilk supply issues, which was a common concern (Table 2). In part, this involved cooking special foods to increase milk supply (see below), but women also reported relying on their mothers for advice about how to deal with issues of both over- and under-supply, as well as when to introduce weaning foods. Women reported being encouraged by their mothers to keep feeding frequently in the early postpartum period to facilitate letdown and build up supply. One grandmother described a method for heating a glass bottle and then placing it on the nipple, causing the heat to draw out a woman’s milk.

Despite having participated in the care of younger siblings and other children, most Himba women lack critical knowledge about early infant care at the time of their first birth. “I didn’t know anything. I was taught by my mother. She told me to hold the baby like this. Told me how to carry her. She also told me how to get her to sleep.” Another woman reported, “My grandmother, who was helping me, told me everything. She said feed the child, it’s crying. Hold the baby with care. Put the breast in the mouth like this. After feeding, put the baby to sleep.” Himba girls participate in childcare of younger siblings on a regular basis from the time they are 5–6 years old, but they are mainly in charge of holding and watching babies, and they are rarely seen helping to care for newborns.

#### Maternal and Infant Health

Himba women reported receiving critical help from their mothers related to both their own postpartum health and the health of their infants. In an uncomplicated delivery, most women in our sample delivered their babies with their mothers acting as birth assistants, though at times, particularly if a laboring woman is in severe pain or has other complications, other women from the community who are known to be experts are brought in to help. One grandmother describes, “When you see the woman is really paining, you send one of the children in the compound to go and get the midwife. You stay with your daughter until she comes.” Milkfat is used to massage the belly during labor, and to help turn the infant if necessary. After the birth, traditional techniques are used to stem postpartum bleeding, “You make a hole and put in hot stones that you have cooked in the fire and leaves from the Mopane tree (*Colophospermum mopane*), which are known for their antimicrobial properties (Kaarina et al. 2017), and then you pour hot water and the woman sits over the steam with a blanket over her. She should do this every day for at least a week, longer if there is more bleeding.” Mothers and elder daughters are the typical helpers in this task, but if a woman gives birth at her husband’s compound and does not have an older daughter to help, she must do it herself. Asked if a woman from her husband’s compound might help in this circumstance, a woman replied, “Someone might help, but not many.” Other remedies, such as a broth made from the root of an acacia tree, are used to help new mothers regain their strength.

New mothers also rely on their mothers to teach them about infant sleep safety. There was a general awareness about suffocation risks and choking, though none of the respondents had heard about Sudden Infant Death Syndrome (SIDS). “Her head must be above the blanket and at an angle so she doesn’t vomit. Her head should also be uphill and she should be on her back.” Mothers also encouraged their daughters to sit up while breastfeeding during the night, instead of trying to feed lying down. “She told me to put the baby like this [holding baby upright]. Sit down, don’t lie. You might lie on him and suffocate him.”

#### Maternal Energy Balance

The support that Himba women receive during the perinatal period affects both energy intake and expenditure. Many women reported that their mothers assisted them with laborious activities such as collecting water and firewood. When women return to their natal compounds, their needs can also be subsidized by help from other female kin in the household (sisters, cousins, aunts). If women were to stay in their marital compound, co-wives might help, but this appears to be dependent on how close the co-wives are.

Mothers also often go above and beyond typical food preparation to ensure that their daughters are well-fed. As one woman reports, “My mother told me that I must eat enough. Maize meal with sour milk. She also slaughtered a goat for me to have meat so that I would make more milk.” Linking provisioning to milk production was mentioned by several of the women who reported that they had milk supply issues. Other women spoke about their first few days postpartum, when their mothers cooked special food for them: “My mom killed a goat for me to eat the meat and make a broth. Then I had milk.”

## Discussion

Grandmothers have long been touted as one among a suite of important alloparents in the human cooperative breeding system (Hrdy 2005; Kramer 2010). But despite their prominence in the literature, data demonstrating positive effects of grandmaternal support is somewhat equivocal. For food sharing and intergenerational transfers, grandmothers in some places play a critical role (Hawkes et al. 1997; Hooper et al. 2015), but in others their efforts are outweighed by other helpers (Kramer 2005; Kramer and Veile 2018). Similarly, the amount of direct care provided to infants and toddlers by grandmothers, compared with other allomothers, is quite variable (Kramer 2010). Despite this disparate evidence, reviews demonstrate strong and consistent patterns showing an association between presence of the maternal grandmother and improved child survival (Fox et al. 2010; Sear and Mace 2008). Taken together, these lines of evidence suggest that supplemental provisioning and childcare are just two pieces of the grandmothering puzzle. Other elements of the grandmaternal niche remain to be systematically investigated. Here we emphasize how multifaceted perinatal support has the potential to improve the health and well-being of mothers and their babies, not only expanding our understanding of grandmothers but bridging to other constructs of human evolution such as the role of emotional support and perinatal care during the difficult childbirths and challenging transitions to motherhood experienced by humans (Trevathan 2017).

### Social Learning in the Perinatal Period

Here we explored the perinatal period embedded in the context of subsistence nutrition, disease ecology, energy balance, kin networks, traditional knowledge, and maternal-infant dynamics atypical of WEIRD populations (Henrich et al. 2010). Populations characterized by predominant subsistence activities and traditional sociocultural practices reveal aspects of adaptive reproductive ecology often disrupted in industrialized contexts. Importantly though, no present-day subsistence culture or population represents the human past nor exclusively occupies the environments in which humans emerged (Crittenden and Schnorr 2017; Lee 1992). From an infant’s perspective, however, the “normalized” breastfeeding dynamic of the mother-infant dyad represents the adaptively relevant environment in which the human neonate evolved. Our findings about breastfeeding difficulties and other maternal anxieties counter widely held perceptions, expectations, and attitudes suggesting that breastfeeding and infant care are intrinsic to womanhood, easily learned through exposure and observation, and only become difficult in contexts where these pathways are disrupted.

Among Himba, breastfeeding is “normalized”; breastfeeding initiation is universal and sustained for many months, nursing is on demand, and clothing does not cover breasts so nursing is highly visible. This is the cultural context in which Himba grow up. But despite this normalization and ubiquity of breastfeeding, Himba women often reported struggling in the early postpartum period following their first births. The women we interviewed spontaneously described difficulties typically encountered by women living in industrialized settings, including nipple/breast pain, difficulty with latch, and concerns about insufficient milk production (Bergmann et al. 2014; Lamontagne et al. 2008; Waller 1946; Williamson et al. 2012). While their struggles are similar, we argue that the consistent, multifaceted support and teaching that Himba grandmothers provide during the first weeks after a birth seems to have an appreciable impact on women’s ability to overcome these difficulties, going on to successfully breastfeed for months and years to come. Similarly, Himba women’s descriptions of the fear and anxiety they experienced during the early postpartum period and their lack of knowledge about basic infant care (e.g., how to hold an infant) further counter common tropes about motherhood in its “natural state.” Again, the main difference appears to be one of timescale. The consistent support that Himba women receive during the perinatal period helps them to overcome difficulties and quell their anxieties in a matter of days, whereas it is not uncommon for women in WEIRD settings to struggle for weeks, or even months, after a birth.

The rapid learning curve that Himba women describe, combined with an intensive period of perinatal co-residence and support, leads us to suggest that social learning from grandmothers and other female kin may be critical to facilitating women’s ability to successfully breastfeed and provide infant care. In some cases, Himba grandmothers were reported to provide very direct instruction. Women reported that their mothers physically and gesturally showed them how to position their babies for improved latch, provided detailed guidance on how often to feed, and explained techniques to protect infants, such as putting them to sleep on their backs and to nurse sitting up rather than lying down. Others described techniques that have presumably been developed and fine-tuned over generations, such as the postpartum steam “bath” described above, or knowledge about which foods and herbs serve as lactogogues. Our findings mirror those of other studies that have similarly shown how the presence of cultural practices and beliefs, acquired through social learning, can positively impact reproductive health. For example, several studies of pregnancy food taboos have been shown to map closely onto species that pose particular dangers to pregnant women and their fetuses (Henrich and Henrich 2010; McKerracher et al. 2016; Placek et al. 2017). Other studies have shown the importance of ideational factors, shared within groups and socially learned, to the practice of early infant care (Hadley et al. 2010; Wutich and McCarty 2008).

Further study might enhance our understanding of perinatal care as a case of social learning and answer questions we are unable to address with our current data. For example, we rely here on self-reports, which have the potential for bias and are not useful for quantifying behavior beyond coarse categories. Observational data would provide exceptional insight into the prevalence of teaching and learning behaviors and would be less subject to bias; however, such data would be very difficult to obtain. The scenarios we describe take place in intimate settings, often during the night in a private sleeping space. Nighttime observations are notoriously rare in time allocation data and present logistical and ethical challenges (Scaglion 1986). Other, less direct types of data could speak to some of these issues. For example, repeated measures of infant health and maternal pain and anxiety could track the effectiveness of grandmothers’ help, particularly in a case-control design comparing women who are and are not co-resident with their mothers after a first birth. Focal follows that concentrate on the first week or two after a birth (particularly first births) would also be extremely useful, as this would increase the chances of capturing otherwise rare instances of potential teaching and provide greater insight into how new mothers learn to breastfeed and care for their infants.

### Integrating Grandmothers within Formal Institutions for Perinatal Care

Difficulties breastfeeding have been most extensively described among Western and high-income populations and can contribute to cessation of breastfeeding ahead of public health recommendations (Bergmann et al. 2014). Across global health settings, medical support and guidance for breastfeeding is now incorporated into more medical specialties, although the clinical management of breastfeeding can be highly variable across hospitals and communities (Breastfeeding Expert Work Group ACOG 2016; Garner et al. 2016; Sriraman and Kellams 2016). Randomized, controlled experimental studies have demonstrated that targeted lactation counseling and clinical support improve breastfeeding outcomes (Renfrew et al. 2012). The medicalization of breastfeeding in part reflects important public health motivations to reverse the precipitous declines in breastfeeding initiation and lactation duration in the mid-twentieth century, which saw breastfeeding rates drop to below 25% for any breastfeeding during hospital stay after parturition and < 6% for any breastfeeding at 6 months of infant age (Ryan et al. 2002). Importantly, the multidecade decline in breastfeeding initiation and duration eroded culturally transmitted knowledge about breastfeeding practices and troubleshooting among women’s kin and friend networks. Indeed, such knowledge could be abandoned or lost from entire familial lineages. This cultural gap in part necessitates expanded lactation education prenatally and clinical lactation support postnatally. In the absence of sufficient postnatal clinical care, mothers often value online social communities for experience-based guidance (Barkhuus et al. 2017), but such resources are not available for millions of mothers. In the Global South, women’s kin, especially their mothers, play an important role in establishing and promoting breastfeeding practices. An epidemiological meta-analysis revealed that, cross-culturally, grandmothers appreciably contributed to breastfeeding decisions (Negin et al. 2016). Importantly though, only 13 of 586 articles met the criteria for inclusion in the meta-analysis, highlighting the limited quantitative investigation of grandmaternal contributions to breastfeeding decisions and lactation performance (Negin et al. 2016). Transformative research in maternal and child health requires a more culturally informed developmental science (Keller 2013; Keller et al. 2005; Yovsi and Keller 2003) that better interprets human phenomena (Kline et al. 2018). This ethnographic case study of Himba perinatal experiences joins a small, but growing body of literature integrating the fields of human behavioral ecology and public health (Martin 2017; Martin et al. 2016; Veile et al. 2014).

Global health initiatives around Safe Motherhood, the Millenium Development Goals, and the Sustainable Development Goals have increased the emphasis on institutionally based births attended by skilled health personnel, designed to reduce maternal and neonatal mortality (Alkema et al. 2016; Jiang et al. 2016; Say et al. 2014). These maternal and child health advances are to be celebrated, and continued improvements should be implemented. Toward that aim, we posit that grandmothers have played an important role during the postpartum period, and understanding that role is vital both to placing birth in its evolutionary context for understanding human life history evolution, as well as to motivate how best to design and implement postpartum health policies that better integrate family into more formalized medical care where culturally relevant. Public health campaigns, such as the First 1000 Days, prioritize infant health but typically overlook concurrent gaps in maternal perinatal and postnatal care (Tully et al. 2017). The prenatal emphasis in Western medicine seemingly recapitulates maternal as vessel, catastrophically neglecting care and respect during labor and delivery as well as deficits in postnatal care (Sadler et al. 2016; Miller et al. 2016; Tully et al. 2017). Cross-culturally, traditional practices emphasize mother-directed care in the postnatal period, most notably recognizing a bounded postpartum period during which mothers are protected, secluded, buffered, assisted, and socio-emotionally supported and ritually recognized (Stern and Kruckman 1983; Eberhard-Gran et al. 2010). Contrasts between advice from kin and advice from medical providers typically and understandably elevate skilled medical knowledge (Kamal 1998), but juxtapositions that place these groups in conflict are risky. Although the World Health Organization and UNICEF perinatal guide for midwives and doctors acknowledges maternal agency, familial support networks, and cultural values (2017), cultural practices often continue to be poorly integrated, risking alienation of patients, clandestine implementation of potentially dangerous traditional practices, and more generally a missed opportunity for knowledge exchange that may become critical when skilled medical providers are unavailable in the context of humanitarian or environmental crises (Chi and Urdal 2018).

## Conclusion

Better recognition of grandmothers as collaborators in maternal and child health may afford more harmonious and effective clinical practice and public health programs in many populations. Grandmaternal care represents a rare behavioral variant within the animal kingdom and is particularly elaborated within human reproductive ecology. Further, among humans, social learning, communication, and lifelong maintenance of consanguineous relationships uniquely position grandmothers to contribute essential perinatal information. Continued systematic research on the ultimate function and proximate contributions of grandmothers, and incorporation of these findings into the medical and public health fields, presents new opportunities for enhancing maternal and child health.

## Footnotes

1 Events described here occurred during fieldwork in Omuhonga (15 km from Okangwati, Namibia) in 2014.
